# Hemiarthroplasty vs. internal fixation for nondisplaced femoral neck fracture in mainland China: a cost-effectiveness analysis

**DOI:** 10.3389/fsurg.2024.1437290

**Published:** 2024-08-29

**Authors:** Shengchun Wang, Lingjie Tan, Bin Sheng

**Affiliations:** Department of Orthopaedics, Hunan Provincial People's Hospital (The First Affiliated Hospital of Hunan Normal University), Changsha, China

**Keywords:** cost-effectiveness analysis, femoral neck fracture, hemiarthroplasty, internal fixation, Markov decision model

## Abstract

**Objective:**

Nondisplaced femoral neck fractures constitute a substantial portion of these injuries. The optimal treatment strategy between internal fixation (IF) and hemiarthroplasty (HA) remains debated, particularly concerning cost-effectiveness.

**Methods:**

We conducted a cost-effectiveness analysis using a Markov decision model to compare HA and IF in treating nondisplaced femoral neck fractures in elderly patients in China. The analysis was performed from a payer perspective with a 5-year time horizon. Costs were measured in 2020 USD, and effectiveness was measured in quality-adjusted life-years (QALYs). Sensitivity analyses, including one-way and probabilistic analyses, were conducted to assess the robustness of the results. The willingness-to-pay threshold for incremental cost-effectiveness ratio (ICER) was set at $11,083/QALY following the Chinese gross domestic product in 2020.

**Results:**

HA demonstrated higher cumulative QALYs (2.94) compared to IF (2.75) but at a higher total cost ($13,324 vs. $12,167), resulting in an ICER of $6,128.52/QALY. The one-way sensitivity analysis identified the costs of HA and IF as the most influential factors. Probabilistic sensitivity analysis indicated that HA was more effective in 69.3% of simulations, with an ICER below the willingness-to-pay threshold of $11,083 in 58.8% of simulations.

**Conclusions:**

HA is a cost-effective alternative to IF for treating nondisplaced femoral neck fractures in elderly patients in mainland China.

## 1 Introduction

As the population ages, hip fractures present a heavy burden for surgeons, health care systems and society. The total number of hip fractures is estimated to exceed 6 million by 2050 (1). In the United States, the annual cost related to hip fractures is estimated to reach $25 billion by 2025 (2). Several studies from mainland China have also demonstrated an increasingly high prevalence of hip fractures (3, 4). Nondisplaced or impacted femoral neck fractures constitute a substantial portion of these hip fractures, accounting for approximately 15% of the overall hip fracture burden (5). The optimal treatment strategy for these nondisplaced femoral neck fractures in the elderly, whether internal fixation (IF) or hemiarthroplasty (HA), remains debated.

Internal fixation using screws is a minimally invasive surgical procedure that offers advantages such as reduced blood loss, shorter operative times, and lower implant costs compared to arthroplasty procedures. However, multiple studies have reported high failure rates for IF, with up to 40% of patients requiring reoperation due to non-union (6), osteonecrosis, or other complications (7–10). Revision for failed fixation is also associated with twice the cost of primary surgery and generally results in poorer clinical outcomes (11). The need for additional surgeries is a major concern in the elderly population, as it can further increase morbidity, mortality, and healthcare utilization. HA allows for earlier weight-bearing and rehabilitation without relying on fracture healing, which can potentially reduce complications such as pneumonia, deep vein thrombosis, and urinary infections. While HA is generally associated with higher initial procedural costs, it may offer long-term benefits in terms of functional outcomes and reduced revision rates. When considering the costs of revision surgeries, some studies found no significant differences between HA and IF (12, 13).

Since femoral neck fractures place a burden on both patients and healthcare resources, clinical outcomes and costs of treatments should be investigated simultaneously to ensure comprehensive decision-making. Economic analyses, especially cost-effectiveness analyses, are usually recommended to assist doctors and policymakers help doctors and policymakers to evaluate the balance between costs and benefits. Cost-effectiveness analysis focuses on the net cost divided by changes in clinical effectiveness, including survivorship, reduced revision rates and quality-adjusted life-year (QALY), which reflects both the quality and quantity of life. Waaler et al. performed a cost-effectiveness analysis based on data from their randomized controlled trial and found that HA was associated with higher QALYs and lower total costs for elderly patients with femoral neck fractures compared to IF (14). Similarly, Yong et al. also reported that HA could provide better outcomes at lower cost (5)**.** However, Liu et al.'s retrospective analysis indicated that the QALY improvements from HA over IF were insufficient to justify the higher costs of HA. They concluded that IF might be a more cost-effective procedure (15).

Given the uncertainty of published results on the cost-effectiveness of HA, it is critical to estimate whether the incremental expenditures incurred by HA could be equated with its better outcomes. There are few health economic studies about this topic in mainland China. We performed this cost-effectiveness analysis using the Markov decision model to compare the cost-effectiveness of HA vs. IF for the treatment of nondisplaced femoral neck fractures in the elderly in the context of China.

## 2 Materials and methods

### 2.1 Overview of the study

The present study was conducted following the guidelines of the Panel on Cost-Effectiveness Analysis in Health and Medicine (16). A payer perspective was employed to evaluate costs and effectiveness. Costs were measured in 2020 United States Dollars (USD, $), and effectiveness was measured in QALYs. On the QALY scale, zero represents death and one represents full health, with lower QALYs indicating time spent with impaired physical and emotional function. We assessed cost-effectiveness by comparing the incremental cost-effectiveness ratio (ICER) to the willingness-to-pay (WTP) threshold. The ICER is calculated by the incremental costs to gain an incremental QALY, which is, in mathematical terms, the difference in costs between two procedures divided by the difference in utility, and can be expressed as Δ Costs/Δ Utility. The WTP threshold represents the maximum amount a patient or policymaker is willing to pay for an additional QALY (17). A procedure is considered cost-effective if its ICER is below the WTP threshold (Supplementary 1). If the procedure is associated with lower costs and higher QALYs, such a procedure is deemed dominant (18). As there are few studies on the WTP thresholds in China, we used the World Health Organization's recommendation to set the WTP threshold at 1–3 times the gross domestic product per QALY (19). In our analysis, the WTP threshold was set at $11,083 in accordance with the 2020 Chinese gross domestic product (Chinese Yuan 71,489) and the exchange rate of 6.45 Chinese Yuan per USD (20). A theoretical cohort of 80-year-old patients with femoral neck fractures was established for the reference case analysis.

### 2.2 Model design

**Figure 1:**
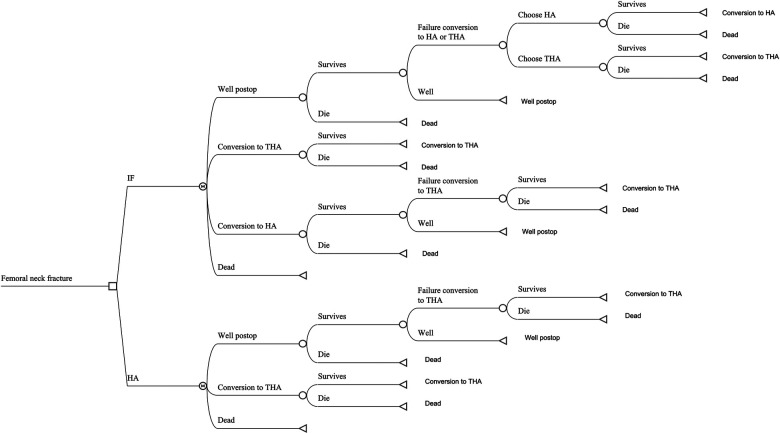
The Markov model for patients with femoral neck fracture. Each patient received internal fixation or hemiarthroplasty. If a patient survived the perioperative period, that patient would stay in the status of successfully postoperative state, experience failure of prior surgery requiring conversion treatment or die from other causes. IF, internal fixation; HA, hemiarthroplasty; THA, total hip arthroplasty.

A Markov decision model was constructed using decision analysis software (TreeAge Pro 2019; TreeAge Software, Williamstown, MA) to compare two strategies: HA and IF. The values for each parameter in the model were obtained from published papers and provided in Table 1 (Supplementary 2). This analysis used a 5-year time horizon with a cycle length of one year. Costs and QALYs were discounted at a rate of 3% per year. The model included four states, categorized as follows: (1) successful IF, (2) successful HA, (3) conversion to total hip arthroplasty (THA) after failed IF or HA, and (4) death. Patients with femoral neck fractures in the model would undergo either IF or HA. After the procedure, surviving patients would either remain in a successful state or experience failure requiring conversion. Among those patients receiving IF, the conversion procedure could be either HA or THA, with an equal probability of 50%. The model tracked patients’ transitions through these states each year. Patients were also exposed to the risks of perioperative death associated with each surgical procedure, which was also incorporated into the model (Figure 1).

**Table 1:** Values of inputs in the markov model.

Parameter	Value	Source (References)
Probability		
Perioperative mortality for IF	4.1%	(21–25)
Perioperative mortality for HA	5.3%	(21–25)
Perioperative mortality for THA	3.9%	(26–29)
Mortality due to other causes	Age-specific mortality	(20)
Annual failure probability of IF	3.3%	(30, 31)
Annual failure probability of HA	1.5%	(32, 33)
Utility (QALY)		
IF	0.63	(10, 15)
HA	0.68	(15, 34, 35)
THA	0.70	(36, 37)
Disutility for salvage treatment	−0.15	(38, 39)
Cost ($)		
IF	8,632	(15)
HA	12,449	(40)
Conversion IF to HA	26,670	(41)
Conversion IF to THA	25,508	(42)
Conversion HA to THA	22,662	(43)
Other		
WTP threshold	11,083	(20)
Discount rate	3%	(44)

IF, internal fixation; HA, hemiarthroplasty; THA, total hip arthroplasty; QALY, quality-adjusted life-year; WTP, willingness-to-pay.

### 2.3 Assumptions

Several assumptions were made in constructing the model: (i) the probability of perioperative death and the mortality of other causes was the same in both HA and IF cohorts; (ii) the failure probability of HA was the same in both cohorts; and (iii) salvage treatment of THA would not fail during the 5-year time-horizon.

### 2.4 Probabilities

The perioperative mortality rates for IF, HA, and THA procedures were 4.1% (21–25), 5.3% (21–23), and 3.9% (26–29), respectively, according to published articles. The mortality rate due to other causes was assumed to follow the age-specific mortality rate obtained from the China Population Census 2020 (20). The failure probability for each procedure was estimated based on studies involving elderly patients aged 80; these values were similar to previously published data and register data (45, 46).

### 2.5 Health utility (effectiveness)

The utilities for HA and IF were based on a recent cost-effectiveness analysis in China by Liu et al. (15). They used the EuroQol 5-dimension index scores to calculate QALY. Previous published data were also aggregated. A disutility of −0.15 (QALY loss) was assigned to the conversion/revision procedure.

### 2.6 Costs

The costs of the HA and IF procedures in China were set at $12,449 and $8,632 (15, 40), respectively, according to Liu et al.'s cost-effectiveness analysis (15). The costs of the conversion procedure (IF to HA, IF to THA, or HA to THA) could not be obtained from Chinese data due to the paucity of published papers. They were estimated based on data published from other countries: IF to HA, $26,670 (41); IF to THA, $25,508 (42); and HA to THA, $22,662 (43).

### 2.7 Sensitivity analysis

We conducted the analysis for the base case at first. All inputs were estimated using a one-way sensitivity analysis, with the range of variation for all variables set to fluctuate by ±20%. The tornado diagram was plotted to visualize these variations. The thresholds for the cost, probability and utility for HA to be considered cost-effective and dominant were calculated and presented.

Probabilistic sensitivity analysis with Monte Carlo simulation was used to determine the overall effect of uncertainty parameters (Table 2). The distribution of each variable was determined by its mean and standard deviation, or set as 10% of the mean value if the standard deviation was unavailable. The cost-effectiveness acceptability curve was used to identify the proportion of patients with an ICER below the given WTP thresholds.

**Table 2:** Parameters for probabilistic sensitivity analysis with monte carlo simulation.

Parameter	Distribution	*α*	*β*	Mean	SD
Annual failure probability of IF	Beta	96.67	2,832.64	2.7%	0.27%
Annual failure probability of HA	Beta	98.49	6,467.18	1.5%	0.15%
Utility of IF (QALY)	Beta	36.37	21.36	0.63	0.063
Utility of HA (QALY)	Beta	31.32	14.73	0.68	0.068
Utility of THA (QALY)	Beta	29.3	12.56	0.70	0.07
Costs of IF ($)	Gamma	100	0.012 (*λ*)	8,632	863.2
Costs of HA ($)	Gamma	100	0.008 (λ)	12,449	1,244.9
Costs of conversion IF to HA ($)	Gamma	100	0.004 (λ)	22,670	2,267
Costs of conversion IF to THA ($)	Gamma	100	0.004 (λ)	25,508	2,550.8
Costs of conversion HA to THA ($)	Gamma	100	0.004 (λ)	22,662	2,266.2

SD, standard deviation; IF, internal fixation; HA, hemiarthroplasty; THA, total hip arthroplasty; QALY, quality-adjusted life-year.

The probabilities and utilities usually follow a beta (β) distribution, while costs generally follow a gamma (λ) distribution.

## 3 Results

### 3.1 Base case

For the base case, HA had a higher cumulative quality of life (2.94 QALYs) compared with IF (2.75 QALYs) at a higher total cost ($13,324 vs. $12,167), yielding an ICER of $6,128.52/QALY. The rate of well-state was 85.0% and 83.8% in the HA and IF cohorts, respectively.

### 3.2 Sensitivity analysis

**Figure 2:**
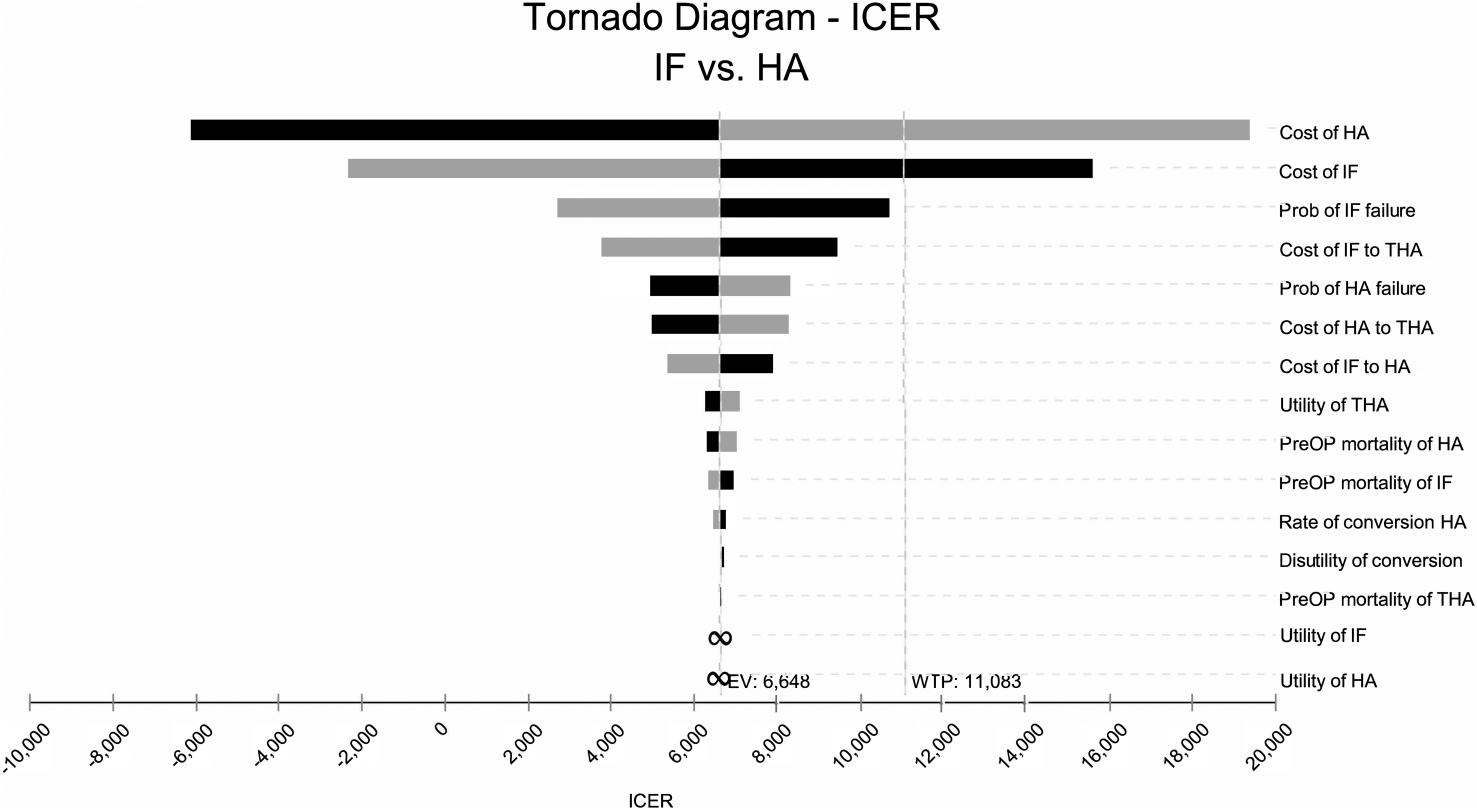
Tornado diagram that contained the one-way sensitivity analysis for each input in the model for internal fixation (IF) versus hemiarthroplasty (HA). The values were individually varied by 20%. The midline represented the bases case with an incremental cost-effectiveness ratio (ICER) of $6,128.52 per quality-adjusted life-year (QALY). The line marked with the willing-ness-to-pay (WTP) threshold indicated that the cost of HA and the cost of IF were the two most sensitive factors. Prob, probability; THA, total hip arthroplasty; PeriOP, perioperative.

**Figure 3:**
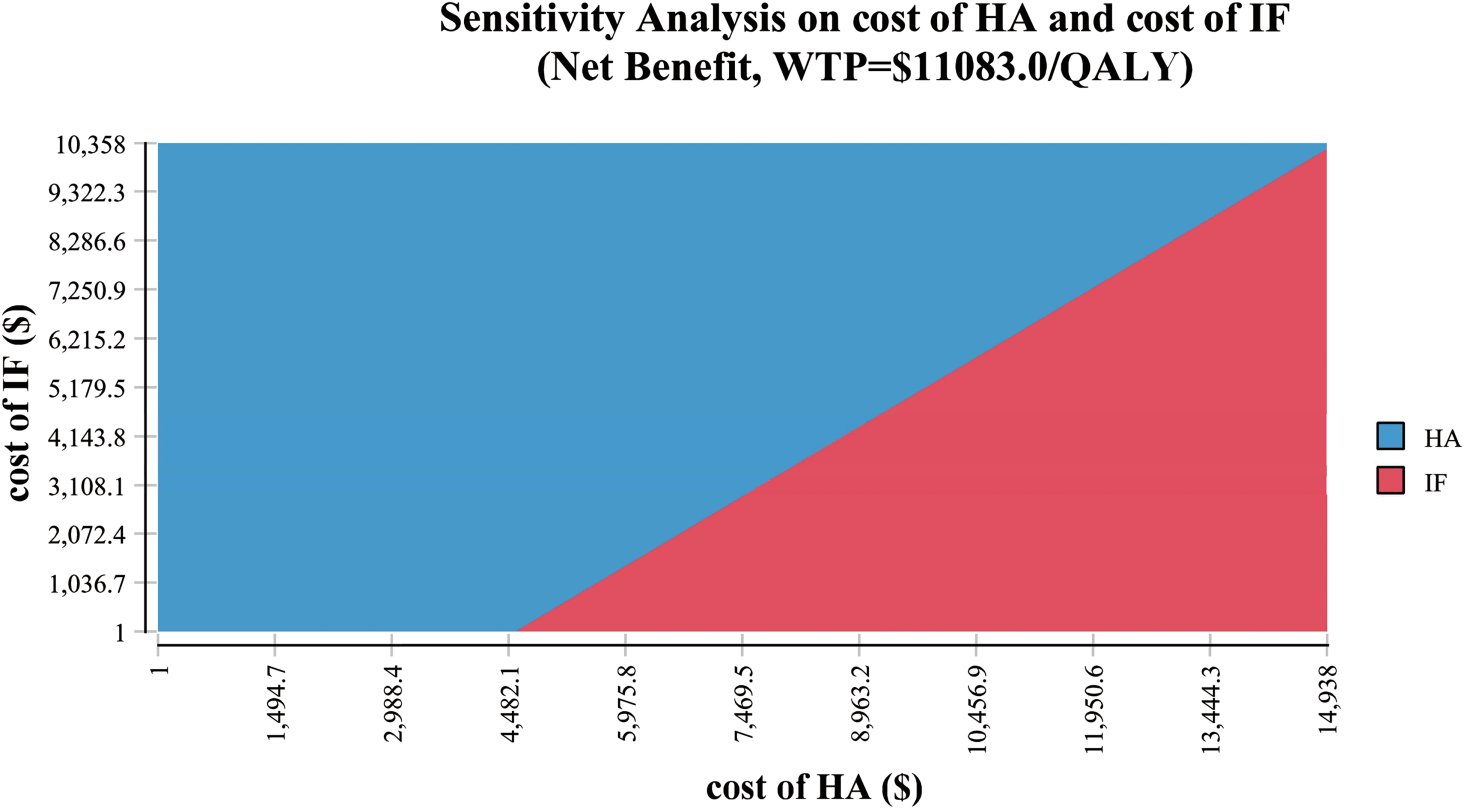
Two-way sensitivity analysis demonstrated the relationship between the cost of hemiarthroplasty (HA) and the cost of internal fixation (IF). The blue area indicated the profiles for which the HA was more cost-effective (the cost was below the willingness-to-pay [WTP] threshold of $11,083 per quality-adjusted life-year [QALY]). The red area indicated that the profiles for which the IF was more cost-effective in the same settings.

The tornado diagram from one-way sensitivity analysis showed that the costs of HA and IF were the two most influential factors on cost-effectiveness within the initially predetermined range (Figure 2). The subsequent two-way sensitivity analysis showed that HA could be more possibly cost-effective when the cost of HA was lower and the cost of IF was higher. If the cost of HA could be lower than $4,482, the HA would be cost-effective even if the IF were a free procedure (Figure 3). The one-way sensitivity analysis also estimated the thresholds of variables for HA to be considered cost-effective and dominant (Table 3).

**Table 3:** Thresholds of parameters for HA to be cost-effective and dominant.

Parameter	Cost-effective	Dominant
Cost of HA ($)	<13,313.9	<11,452
Cost of IF ($)	>7,778	>10,012
Prob of IF failure	>2.8%	>4.3%
Prob of HA failure	<2.4%	<0.4%
Utility of IF (QALY)	<0.648	NA
Utility of HA (QALY)	>0.662	NA

IF, internal fixation; HA, hemiarthroplasty; QALY, quality-adjusted life-year.

**Figure 4:**
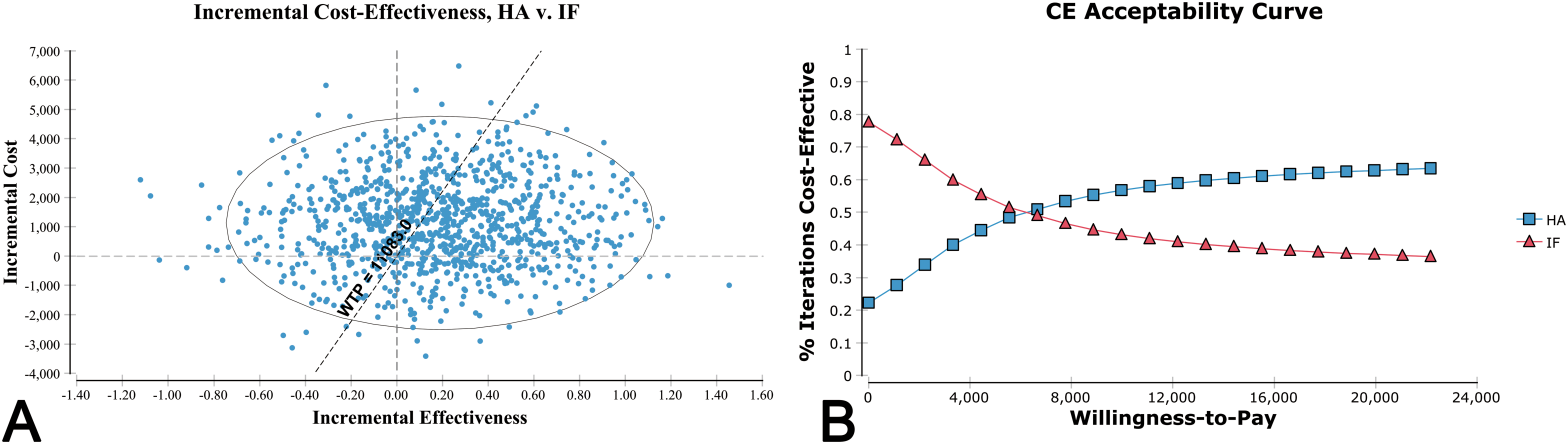
**(A)** Scatter plot of Monte Carlo simulation demonstrated the distribution of 10,000 samples. The ellipse reflected the range of 95% confidence intervals for the cost-effectiveness ratios. The vertical dotted line represented the threshold of incremental effectiveness. The horizontal dotted line represented the threshold of incremental cost. The oblique dotted line represented the willingness-to-pay (WTP) threshold of $11,083 per quality-adjusted life-year (QALY). The samples that were located in the southeast of the WTP line were considered as cost-effective after hemiarthroplasty (HA) compared with internal fixation (IF). **(B)** The cost-effectiveness (CE) acceptability curves depicted the relationship between the WTP threshold (the United States Dollar per quality-adjusted life-year) on the *X* axis and the proportion of samples in Monte Carlo simulation who had an incremental cost-effectiveness ratio below the given WTP thresholds.

In the Monte Carlo simulation analysis, the cost of HA was $13,310 ± 1,189 compared to $12,174 ± 909 for IF. The effectiveness of HA was 2.95 ± 0.74 QALYs, while that of IF was 2.76 ± 0.63 QALYs, resulting in an ICER of $5,983.23/QALY. The scatter plot represented the relationship between the incremental cost and the incremental effectiveness in the probability analysis (Figure 4A). HA was more expensive in 77.6% of the samples in the simulation, yet more effective in 69.3% of the samples. The acceptability curve assessed the uncertainty in ICER by plotting the proportion of the simulations that were cost-effective under specific WTP thresholds (Figure 4B). The curve depicted that 58.8% of HA simulations had an ICER below the WTP threshold of $11,083, and the rate was 64.1% when the WTP threshold was doubled to $22,166.

## 4 Discussion

To the best of our knowledge, this study is the first to utilize a Markov decision model to estimate the cost-effectiveness of HA and IF for treating femoral neck fractures in elderly patients from a payer perspective in mainland China. The primary findings were that HA for an 80-year-old patient with a femoral neck fracture was associated with an ICER of $6,128.52/QALY compared to IF. This ICER was below the primary WTP threshold of $11,083/QALY, suggesting that HA was cost-effective.

The socioeconomic burden of hip fractures is substantial (47–50). Traditional treatments for nondisplaced and valgus-impacted femoral neck fractures involve HA and IF. Although IF is a lower-cost procedure compared to HA, it has been reported to have higher failure rates, introducing significant uncertainty regarding the optimal treatment strategy. Our research found that the HA cohort had a better quality of life than the IF group (2.94 QALYs vs. 2.75 QALYs), with a slightly higher proportion of patients in the “well” state (85.0% vs. 83.8%). In addition, the ICER of HA suggested that the improved function and lower revision rates associated with HA outweighed its higher costs. In a follow-up study with a 2-year period by Frihagen et al. (51), the total costs of HA were even lower than those of IF, despite similar primary treatment costs. This discrepancy could be attributed to the high revision rates associated with IF (52), which was also observed in our study. However, some studies recommended that the main advantages of IF lie not only in its lower costs but also less trauma and lower infection rates (34, 53, 54). Those authors suggested that IF might be more suitable for frail or immobile patients with shorter life expectancies.

Within the given range of 20%, our results indicated that the two most sensitive factors affecting the cost-effectiveness of HA were the costs of the two procedures. Further two-way sensitivity analysis showed that as HA costs decreased or IF costs increased, the cost-effectiveness advantage of HA became more evident. Furthermore, our results reflected that a relatively modest 8% reduction in the initial price of HA could potentially shift it from being a cost-effective option to becoming the dominant treatment strategy over IF. Over a five-year time horizon, HA not only generated higher QALYs but also incurred lower overall costs compared to IF. The insights from our study can be valuable for healthcare policymakers as they seek to balance the trade-offs between treatment costs and patient benefits. By identifying the price point at which HA becomes the dominant strategy, these findings can help to optimize the use of HA in managing elderly femoral neck fractures.

The failure rates of both procedures are also important in estimating the cost-effectiveness. Based on published papers, the failure probability of IF was nearly two times as high as that of HA. The failure rates of IF varied across studies, ranging from 16% to 42% (55–57). Failed IF, often due to non-union of head necrosis, usually required salvage arthroplasty, which carried a substantial economic burden. Besides, hip function after arthroplasty is generally higher than after IF (35, 40, 58, 59), which also increases the relative utility of arthroplasty. Several studies have identified risk factors for the revision after IF, including female gender, smoking and advanced age (>80 years) (60–62). Our results can offer additional information to guide proper treatment choices.

The Monte Carlo probabilistic sensitivity analysis revealed that over two-thirds of patients obtained greater QALYs, with 85% of them (equivalent to 58.8% of all patients) showing an ICER lower than the threshold of $11,083. Given the uncertainty of model variables, the simulation procedure can provide a more realistic estimation of ICER than the one-way sensitivity analysis, with consistent results when compared to our prior analysis.

We acknowledge several limitations in our study. First, due to limited data availability, we relied on published data rather than prospectively collected data to estimate the model inputs. For instance, Liu et al. published a cost-effectiveness analysis using retrospective data with a two-year follow-up (15), and other studies were primarily based on published data (5, 14, 63, 64). Although we conducted sensitivity analyses for variables, the results might be influenced by the reliance on these values. Second, our model only considered the salvage procedure. Other complications, such as infection (65), dislocation, and removal of the fixations, were not incorporated into the model. Third, our model assumed that the conversion procedure to THA would not fail. This assumption was based on a 10-year follow-up study that reported a mere 1.6% failure rate after salvage THA over 10 years (29). With a 5-year time horizon, we can only estimate the early economic outcomes of HA vs. IF. Therefore, the salvage procedures for failed THA, including revision THA, femoral megaprosthesis, and total femoral replacement (66), are not considered in this model. Fourth, the research was conducted using the USD system, facilitated by the exchange rate, to enhance comprehension of the results. However, the generalizability of the research findings might be limited due to variations in national or regional healthcare systems.

## 5 Conclusions

Our study supported that HA for the elderly patients with femoral neck fractures was a cost-effective alternative to the IF in mainland China.

## Data Availability

The original contributions presented in the study are included in the article/Supplementary Material, further inquiries can be directed to the corresponding author.

## Author's note

All authors had full access to the data in the study and take responsibility for the integrity of the data and the accuracy of the data analysis. The probabilities and utilities usually follow a beta (β) distribution, while costs generally follow a gamma (λ) distribution.
