# Supplementary material for: Hemiarthroplasty vs. internal fixation for nondisplaced femoral neck fracture in mainland China: a cost-effectiveness analysis
Source: Front Surg. 2024 Aug 29;11:1437290. doi: 10.3389/fsurg.2024.1437290 (PMC11391527; doi:10.3389/fsurg.2024.1437290)
Supplement: Supplementary file 1 [file Table1.docx]

Supplementary Material

Hemiarthroplasty versus internal fixation for nondisplaced femoral neck fracture in mainland China: a cost-effectiveness analysis

Shengchun Wang, Lingjie Tan, Bin Sheng^*^

*** Correspondence:** Bin Sheng: hnsrmyyshengbin@outlook.com

1. **Clarification of quality-adjusted life-years (QALYs), incremental cost-effectiveness ratio (ICER) and willingness-to-pay (WTP)**

QALYs takes into account both the quantity and quality of extra life. It is a main part of “health status measurements”. On the QALY scale, 0 represents death and 1 represents full health, with lower QALYs indicating time spent with impaired physical and emotional function. In the cost-effectiveness analysis, it is usually employed as the measurement of health utility. Usually, the value of QALY is estimated based on quality-of-life evaluation methods, such as EuroQol 5-dimension (EQ-5D), SF-36 and so on. For instance, a patient survives 3 years with the health utility of 0.5, the quality of life during this period is 0.5*3 = 1.5 QALYs. This patient experiences the equivalent of full health for 1.5 years.

ICER is calculated by the incremental costs to gain an incremental QALY. The calculation of ICER is very important in the cost-effectiveness analysis. It can be expressed in the following (1)


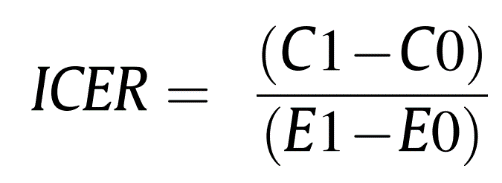
(1)

C_1_ and E_1_ represent the cost and effectiveness (health utility) in procedure 1, and C_0_ and E_0_ represent the cost and effectiveness in procedure 0 (reference) to be compared to procedure 1. If the ICER is low (close to zero), it suggests that the additional cost of procedure 1 (C1) is similar to the reference procedure (C0), but the effectiveness (E1) is significantly higher.

WTP threshold represents the maximum amount a patient or policymaker is willing to pay for an additional QALY. When comparing two procedures, the intervention is considered cost-effective if ICER ≤ WTP. Generally, WTP threshold should be investigated by survey or interview. If WTP threshold is not well established, World Health Organization recommends the WTP threshold to be set at 1-3 times the gross domestic product per QALY.

1. **Meta-analysis method to identify published papers**

The PubMed database was searched up to 1st March 2023. The search keywords were: (femoral neck fracture) AND (hemiarthroplasty) AND (internal fixation) OR (screw fixation) AND (China OR Chinese). MeSH Terms were also used. Eligible studies were screened by the following inclusion criteria: (1) femoral neck fractures in the elderly; (2) reporting the cost and effectiveness of hemiarthroplasty or internal fixation, including QALY or health status measurements which could be transferred to QALY; (3) in the context of China. We excluded non-English language reports, in vitro researches, case reports, brief reports, conference abstract/posters or reviews. After the removal of duplicates, two authors independently reviewed the titles and abstracts to screen for potentially eligible studies. Full-texts were then assessed independently by the same two reviewers to identify the final list of publications.
